# Lack of Genetic Interaction between *Tbx18* and *Tbx2/Tbx20* in Mouse Epicardial Development

**DOI:** 10.1371/journal.pone.0156787

**Published:** 2016-06-02

**Authors:** Franziska Greulich, Carsten Rudat, Henner F. Farin, Vincent M. Christoffels, Andreas Kispert

**Affiliations:** 1 Institut für Molekularbiologie, Medizinische Hochschule Hannover, Hannover, Germany; 2 Department of Anatomy, Embryology and Physiology, Academic Medical Center, University of Amsterdam, Amsterdam, The Netherlands; Northwestern University, UNITED STATES

## Abstract

The epicardium, the outermost layer of the heart, is an essential source of cells and signals for the formation of the cardiac fibrous skeleton and the coronary vasculature, and for the maturation of the myocardium during embryonic development. The molecular factors that control epicardial mobilization and differentiation, and direct the epicardial-myocardial cross-talk are, however, insufficiently understood. The T-box transcription factor gene *Tbx18* is specifically expressed in the epicardium of vertebrate embryos. Loss of *Tbx18* is dispensable for epicardial development, but may influence coronary vessel maturation. In contrast, over-expression of an activator version of TBX18 severely impairs epicardial development by premature differentiation of epicardial cells into SMCs indicating a potential redundancy of *Tbx18* with other repressors of the T-box gene family. Here, we show that *Tbx2* and *Tbx20* are co-expressed with *Tbx18* at different stages of epicardial development. Using a conditional gene targeting approach we find that neither the epicardial loss of *Tbx2* nor the combined loss of *Tbx2* and *Tbx18* affects epicardial development. Similarly, we observed that the heterozygous loss of *Tbx20* with and without additional loss of *Tbx18* does not impact on epicardial integrity and mobilization in mouse embryos. Thus, *Tbx18* does not function redundantly with *Tbx2* or *Tbx20* in epicardial development.

## Introduction

The epicardium is an epithelial monolayer that completely covers the outer surface of the heart. It protects the underlying myocardium and allows mobility of the heart within the pericardial cavity. In addition to this structural role in homeostasis, the epicardium has been recognized as an important source of cells and signals directing and modulating myocardial growth and vascularization both in development and under injury conditions (for recent reviews see [1, 2]).

Epicardial development in the mouse starts at embryonic day (E) 9.5 with the formation of the proepicardium, a cauliflower-like mesothelial cell aggregate at the venous pole of the heart [3, 4]. Cells of the proepicardium delaminate and attach to the adjacent myocardium. At E10.5, a contiguous epithelial epicardial layer surrounds the heart tube. Between E11.5 and E14.5, individual epicardial cells undergo an epithelial-mesenchymal transition (EMT), invade the underlying myocardium and largely differentiate into smooth muscle cells (SMCs) and cardiac fibroblasts [5–10]. Concomitantly, the epicardium acts as a source of signals that nurture the myocardium and promote the in-growth of the coronary plexus and vascularization of the cardiac muscle [10, 11]. Intriguingly, it has been uncovered in recent years both in zebrafish and mouse that the adult epicardium can reactivate an embryonic gene program upon injury conditions [12]. As a consequence, the epicardium secretes factors that promote neovascularization of the myocardium, and provides cells that upon differentiation into fibroblasts and SMCs contribute to scar formation [13, 14].

Although several signaling pathways and transcription factors have been implicated in the distinct subprograms of epicardial development, namely proepicardium formation, epicardial EMT, fate decision or epicardial-myocardial crosstalk [11, 15], we are far from understanding the tight regulatory networks orchestrating all of these processes in time and space, and using them for regenerative purposes.

T-box (*Tbx*) genes encode a large family of transcription factors that regulate a variety of developmental processes in both vertebrates and invertebrates. They are characterized by a common DNA-binding motif, the T-box that recognizes and binds conserved DNA-elements in the genome to mediate transcriptional activation and/or repression of target genes. T-box genes often act in a combinatorial or hierarchical fashion and frequently exhibit an exquisite dose-sensitivity (for reviews see [16, 17]). In the developing mammalian heart, expression of six of the 17 murine family members have been detected and related to different subprograms of myocardial patterning and differentiation (for a review see [18]). *Tbx5* and *Tbx20* act in the early heart tube, and independently activate the chamber myocardial gene program [19–23] whereas *Tbx2* and *Tbx3* act together to locally repress this program to favor valvuloseptal and conduction system development [24–26]. *Tbx1* acts in the pharyngeal mesoderm to maintain proliferation of mesenchymal precursor cells for formation of a myocardialized and septated outflow tract [27]. *Tbx18* is expressed in the sinus venosus region at the posterior pole of the heart and is required for myocardialization of the caval veins and formation of a large portion of the sinoatrial node [28, 29].

Additional roles of these *Tbx* genes in epicardial development have been suggested. *Tbx5* expression was detected in a heterogenous fashion in the proepicardium at E9.5 and the nascent epicardium at E10.5. Epicardial expression strongly declined after this stage. Conditional deletion of *Tbx5* from the (pro-)epicardium led to reduced attachment of proepicardial cells to the myocardium and epicardial blebbing that are probably causative for the reduced epicardial EMT, fibroblast and SMC formation, and defective myocardial and coronary vessel maturation [30].

*Tbx18* is strongly expressed in the proepicardium at E9.5 and is maintained in the epicardium until birth in all vertebrate models analyzed to date [9, 31–34]. We have recently reported that *Tbx18*-deficient mice that were maintained on an outbred background do not exhibit epicardial defects whereas mice with epicardial overexpression of an activating form of TBX18 (a VP16-fusion protein) show loss of epicardial EMT due to premature SMC differentiation of epicardial cells [35]. Since TBX18 possesses transcriptional repressor activity via an eh1-motif near the N-terminus that recruits Groucho corepressors [36], these findings point to a possible redundancy with another repressing member of this gene family in maintaining the progenitor character of epicardial cells. This hypothesis is supported by a recent study in which mice deficient for another null allele of *Tbx18* exhibit epicardial blebbing and coronary defects when maintained on an inbred background [37].

We here aimed to decipher a functional redundancy of *Tbx18* with other transcriptional repressors of the *Tbx* gene family in epicardial development. We identify *Tbx2* and *Tbx20* as being coexpressed with *Tbx18* in the developing (pro-)epicardium and subsequently test for genetic interaction of *Tbx18* and *Tbx2*/*Tbx20* in this tissue.

## Material and Methods

### Ethics statement

All animal work conducted for this study was performed according to European and German legislation. Breeding of mutant mouse lines was approved by the Niedersächsisches Landesamt für Verbraucherschutz und Lebensmittelsicherheit (Permit Number: AZ33.12-42502-04-13/1356, AZ33.12-42502-04-13/1875).

### Mice and genotyping

For the generation of *Tbx18*-deficient embryos, males heterozygous for a *cre* knock-in allele of *Tbx18* (*Tbx18*^*tm4(cre)Aki*s^, synonym:*Tbx18*^*cre*^) [38] were mated to female mice heterozygous for *a LacZ* knock-in allele of *Tbx18* (*Tbx18*^*tm1*.*1Akis*^, synonym: *Tbx18*^*LacZ*^) [39] or a *GFP* knock-in allele of *Tbx18* (*Tbx18*^*tm2Akis*^, synonym: *Tbx18*^*GFP*^) [28]. Female mice homozygous for a floxed allele of *Tbx2* (*Tbx2*^*tm2*.*1Vmc*^, synonym: *Tbx2*^*fl*^) [40] were crossed with *Tbx18*^*cre/+*^*;Tbx2*^*fl/+*^ males to obtain embryos with epicardium-specific loss of *Tbx2*. The reporter allele *Gt(ROSA)26*^*Sortm4(ACTB-tdTomato*,*-EGFP)Luo*^ (synonym: *R26*^*mTmG*^) [41] was combined with the *Tbx18*^*cre*^ line and a *Tbx2*^*cre*^ line (*Tbx2*^*tm1*.*1(cre)Vmc*^) [42] for fate analysis. For the generation of mice compound mutant for *Tbx20*, we used the previously described null allele *Tbx20*^*tm1Akis*^ (synonym: *Tbx20*^*LacZ*^) [20]. Mice with epicardial overexpression of *Tbx2* derived from matings of *Tbx18*^*cre/+*^*;R26*^*mTmG/+*^ males with females homozygous for *Hprt*^*tm2(CAG-TBX2*,*-EGFP)Akis*^ (synonym: *Hprt*^*CAG*::*TBX2*^) [26]. All mice were maintained on an outbred (NMRI) background. Mice were kept with regulated temperature (18–22°C) and humidity (~50%) with a 12 h light/dark cycle. Vaginal plugs were checked in the morning after mating, for timed pregnancies noon was taken as E0.5. Female mice were sacrificed by cervical dislocation. Embryos were harvested in PBS, decapitated, fixed in 4% paraformaldehyde overnight and stored in 100% methanol at -20°C before further use. Genomic DNA prepared from yolk sacs or tail biopsies was used for genotyping by PCR.

### Epicardial explant cultures

Explant cultures of primary epicardial cells were obtained as described before [9].

### RNA isolation, reverse transcription and PCR analysis

RNA of epicardial explants was obtained using PeqGold RNApure (Peqlab) according to the manufacturer’s manual and subsequently transcribed using the RevertAid Reverse Transcriptase (Fermentas). 3 μl of undiluted epicardial cDNA or 1 μl of control cDNA (prepared from different embryonic tissues) were used in the PCR reaction. Primers, PCR conditions and controls are depicted in S1 Table.

### Histological analysis

For histological stainings embryos were fixed in 4% paraformaldehyde overnight, transferred to PBS, paraffin embedded, and sectioned to 5-μm. Sections were stained with hematoxylin and eosin following standard procedures.

### *In situ* hybridization analysis

Nonradioactive *in situ* hybridization analysis with digoxigenin-labeled antisense riboprobes was performed on 10 μm sections of paraffin-embedded embryos as described [43].

### Immunofluorescent detection of proteins

For immunofluorescence analysis on 5 μm paraffin sections anti-TBX3 (1:50, Santa Cruz, sc-31656), anti-TBX2 (1:1000, Millipore, 07–318), anti-TBX18 (1:50, Santa Cruz, sc-17869), anti-TAGLN (1:300, Abcam, ab14106-100), anti-NOTCH3 (1:300, Abcam, ab23426), FITC-conjugated anti-ACTA2 (1:200, Sigma, F3777), anti-EMCN (1:50, obtained from D. Vestweber, MPI Münster, Germany), anti-POSTN (1:300, Abcam, ab14041), anti-WT1 (1:200, Calbiochem, CA1026), anti-GFP (1:200, Roche, 11 814 460 001) or rabbit polyclonal antibody anti-COL type IV (1:200, Millipore, AB756P) were used as primary antibodies. Biotinylated anti-rabbit IgG (1:200, Dianova), biotinylated anti-rat IgG (1:200, Dianova), Alexa488-conjugated anti-mouse IgG (1:200, Invitrogen) and Alexa488-conjugated anti-rabbit IgG (1:200, Invitrogen) were used as secondary antibodies. The signals of all primary antibodies (except anti-GFP, anti-COLIV and anti-ACTA2) were amplified using the Tyramide Signal Amplification (TSA) system from Perkin-Elmer (NEL702001KT, Perkin Elmer). For double staining, the second antigen was stained for after the staining for the first one was finished. Nuclei were stained with 4,6-diamidino-2-phenylindole (DAPI) (Roth).

### Documentation, Quantification and Statistics

Sections were photographed using a Leica DM5000 microscope with Leica DFC300FX digital camera. All images were processed in ImageJ and Adobe Photoshop CS4. Statistical analyses were performed using the 2-tailed Student’s t-test. Data were expressed as mean±SD. Differences were considered not significant when the P-value was higher than 0.05. At least two specimens were analyzed per stage, genotype and assay.

## Results

### Expression of T-box genes during epicardial development

**Fig 1:**
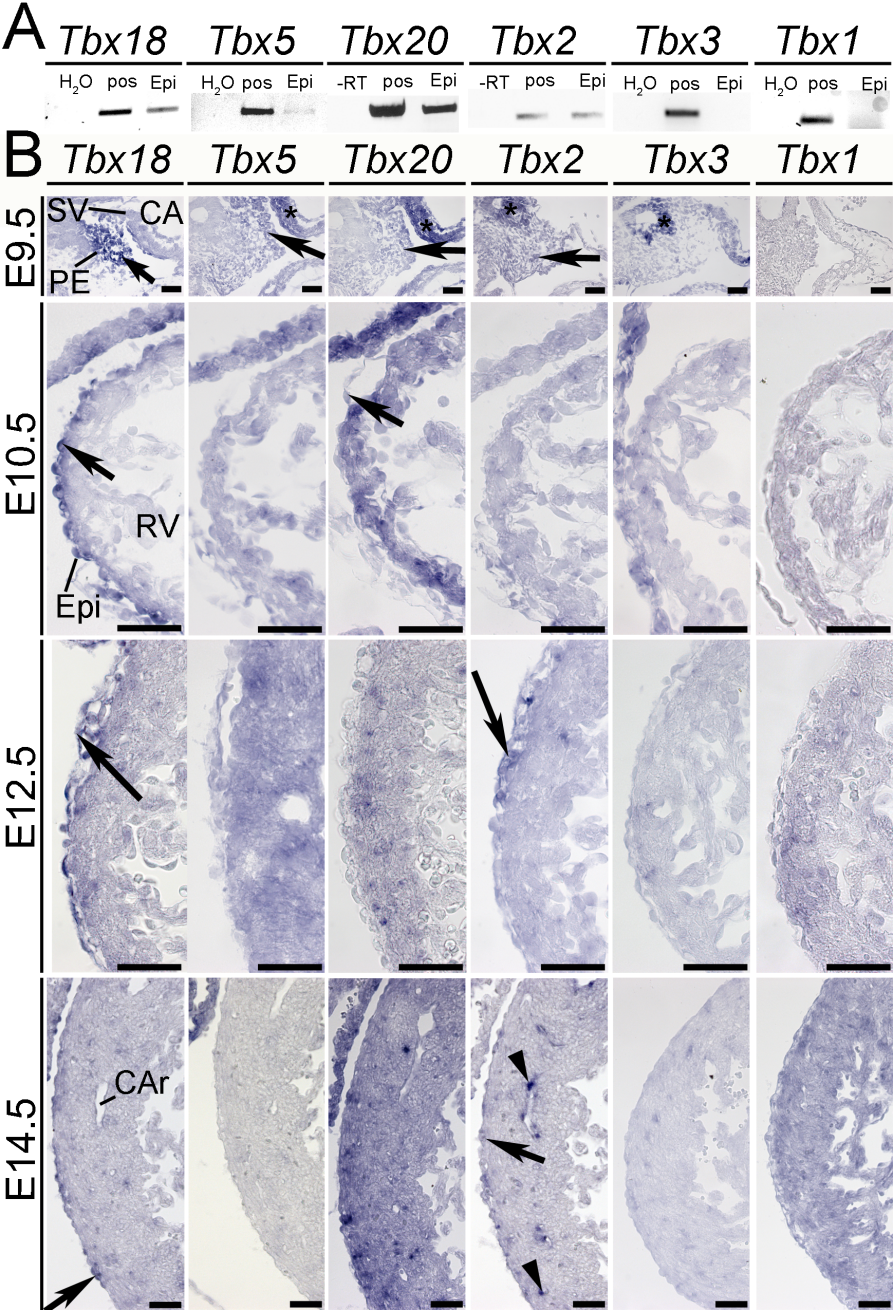
T-box gene expression during epicardial development. (A) Qualitative RT-PCR analysis detects expression of *Tbx18*, *Tbx5*, *Tbx20* and *Tbx2* but not of *Tbx3* and *Tbx1* in undifferentiated epicardial cells from cardiac explant cultures (Epi). 32 epicardial explant cultures were pooled and used for qualitative PCR. H_2_O refers to a negative control without cDNA, pos to a positive control of a tissue with known expression (see S1 Table). (B) *In situ* hybridization analysis of *Tbx18*, *Tbx5*, *Tbx20*, *Tbx2*, *Tbx3* and *Tbx1* expression on sagital (E9.5) and transverse (E10.5, E12.5, E14.5) sections through the heart. Shown are higher magnifications of the proepicardium (E9.5) and of the right ventricle (E10.5 to E14.5). Black arrows indicate proepicardial and epicardial expression of *Tbx18*, *Tbx5*, *Tbx20* and *Tbx2*, asterisks point to known expression domains of *Tbx5* and *Tbx20* in the atrium, and of *Tbx2* and *Tbx3* in the liver primordium at E9.5. Black arrowheads indicate coronary artery expression of *Tbx2* at E14.5. Scale bars are 50 μm. CA, common atrium; CAr, coronary artery; Epi, epicardium; PE, proepicardium; RV, right ventricle; SV, sinus venosus.

To determine whether the members of the cardiac T-box gene family *Tbx1*, *Tbx2*, *Tbx3*, *Tbx5*, *Tbx20* are coexpressed with *Tbx18* in epicardial development, we performed a comparative expression analysis in mouse embryos. As a sensitive assay we first performed qualitative RT-PCR analysis from E11.5 epicardial explant cultures grown for three days under serum-free conditions. This assay revealed the expression of *Tbx18*, *Tbx20*, *Tbx2* and *Tbx5* in undifferentiated epicardial cells whereas *Tbx3* and *Tbx1* were not detected (Fig 1A). To resolve epicardial expression in time and space we subsequently performed section *in situ* hybridization of E9.5 to E14.5 hearts (Fig 1B). At E9.5, expression of *Tbx18* was strong in the proepicardium. Expression of *Tbx20*, *Tbx5* and *Tbx2* was markedly weaker but still above background levels. From E10.5 to E14.5, *Tbx18* expression was detected in the epicardium but not in subepicardial cells in the right ventricle. (Due to endogenous expression of *Tbx18* in the myocardium of the left ventricle and the interventricular septum [44], we restricted our analysis on the right ventricle). Expression of *Tbx5*, *Tbx3* and *Tbx1* could not be detected in the epicardium at any of these stages. In contrast, we found expression of *Tbx20* in epicardial cells at E10.5 (Fig 1B, arrow). Due to the myocardial expression of *Tbx20* (Fig 1B, *), *Tbx20-*expressing epicardial and epicardium-derived cells could not be distinguished by this method. *Tbx2* transcripts were detected in a subset of epicardial cells (Fig 1B, arrow) at E12.5 and at E14.5, and in coronary arteries at E14.5 (Fig 1B, black arrowheads).

**Fig 2:**
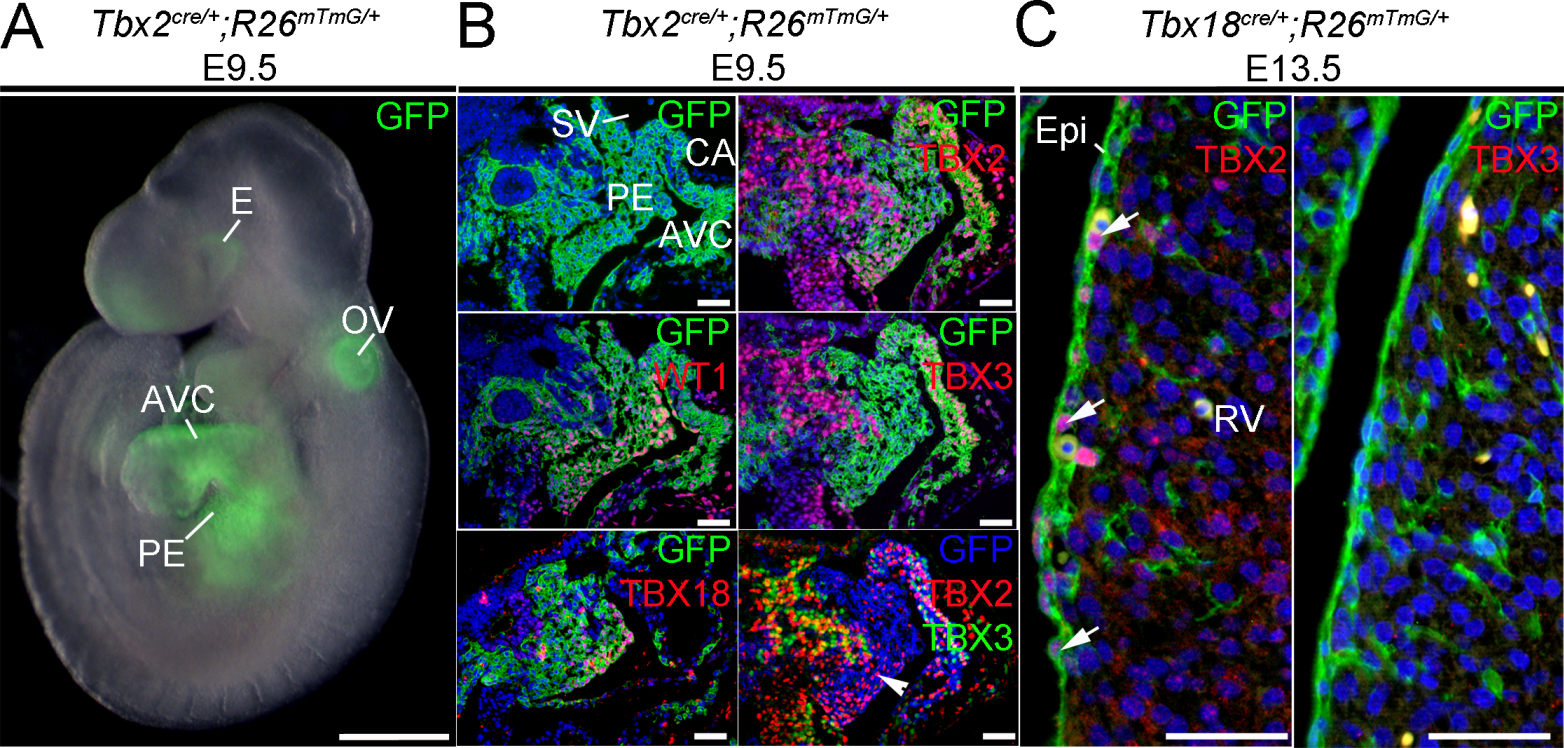
TBX2 is expressed in the proepicardium and epicardium. (A) Epifluorescence of a *Tbx2*^*cre/+*^*;R26*^*mTmG/+*^ embryo at E9.5 reveals the contribution of formerly *Tbx2*-expressing cells to the atrio-ventricular canal (AVC), the otic vesicle (OV), the eye (E) and the proepicardium (PE) (n = 5). The scale bar is 500 μm. (B) Lineage tracing of *Tbx2*-expressing cells on sections of E9.5 embryos by immunofluorescent detection of a GFP reporter and/or the epicardial markers TBX18 and WT1 (left row) confirms the contribution of *Tbx2*-expressing cells to the proepicardium (n = 3). Double immunofluorescence against GFP and TBX2 or TBX3, respectively, (right row) shows expression of TBX2 in the caudal part of the proepicardium. Note that the anti-TBX2 antibody recognizes cells that do not recombine after *cre* expression from the *Tbx2* promoter. The third picture of the lower row shows an *in silico* overlay of the expression domains of TBX2 and TBX3 co-stained with the *Tbx2*-lineage label GFP on neighboring sections. Only TBX2-positive (red), TBX3-negative and GFP-positive (blue) domains relate to *Tbx2* expression domains (white arrowhead). The scale bars are 50 μm. (C) TBX2 but not TBX3 protein was detected by immunofluorescence against TBX2 and TBX3 in epicardial and subepicardial cells of *Tbx18*^*cre/+*^*;R26*^*mTmG/+*^ embryos at E13.5 (white arrows, n = 2). Co-staining against the *Tbx18*-lineage label GFP clearly identifies epicardial and epicardium-derived cells. The scale bars are 50 μm. CA, common atrium; Epi, epicardium; RV, right ventricle; SV, sinus venosus.

For further clarification, we also analyzed TBX2 and TBX3 protein expression by immunofluorescence. At E9.5, Wilms tumor 1 (WT1) and TBX18 were found in the entire proepicardium as previously reported [44, 45] whereas TBX2 protein was confined to the caudal part of this tissue. Lineage analysis using a *Tbx2*^*cre*^ line [42] and a *Rosa26*^*mTmG/+*^ reporter line [41] showed that the proepicardium itself was completely derived from cells formerly expressing *Tbx2* (Fig 2A and 2B). At E13.5, a subset of epicardial and subepicardial cells expressed TBX2 protein as shown by double immunofluorescence against TBX2 and GFP, that in this case marked the epicardial lineage by epicardium-specific recombination under the control of the *Tbx18* promoter [9] (*Tbx18*^*cre/+*^*;R26*^*mTmG/+*^ mice) (Fig 2C). Expression of TBX3 was neither detected in the proepicardium at E9.5 nor in the epicardium at E13.5 (Fig 2B and 2C), confirming as in the case of TBX2 our mRNA expression analysis. We conclude that *Tbx18* is co-expressed with *Tbx5*, *Tbx20* and *Tbx2* in a subset of cells in the proepicardium at E9.5 and in a subset of epicardial cells until E14.5.

### Combined loss of *Tbx18* and *Tbx2* does not affect epicardial development

Since *Tbx2* is co-expressed with *Tbx18* in a subset of proepicardial and epicardial cells and encodes a transcriptional repressor like *Tbx18* [36, 46], we tested for functional redundancy of the two genes in epicardial development by a conditional gene targeting approach. (Pro)-epicardial deletion of *Tbx2* was achieved using a floxed allele of *Tbx2* and the *Tbx18*^*cre*^ mouse line that mediates robust recombination in the proepicardium, and in the epicardium and its descendants [9, 47]. Absence of TBX18 protein in the epicardium/pericardium of *Tbx18*-null embryos, and of TBX2 protein in *Tbx18*^*cre/+*^*;Tbx2*^*fl/fl*^ embryos at E10.5 confirmed the suitability of this genetic approach (S1 Fig).

**Fig 3:**
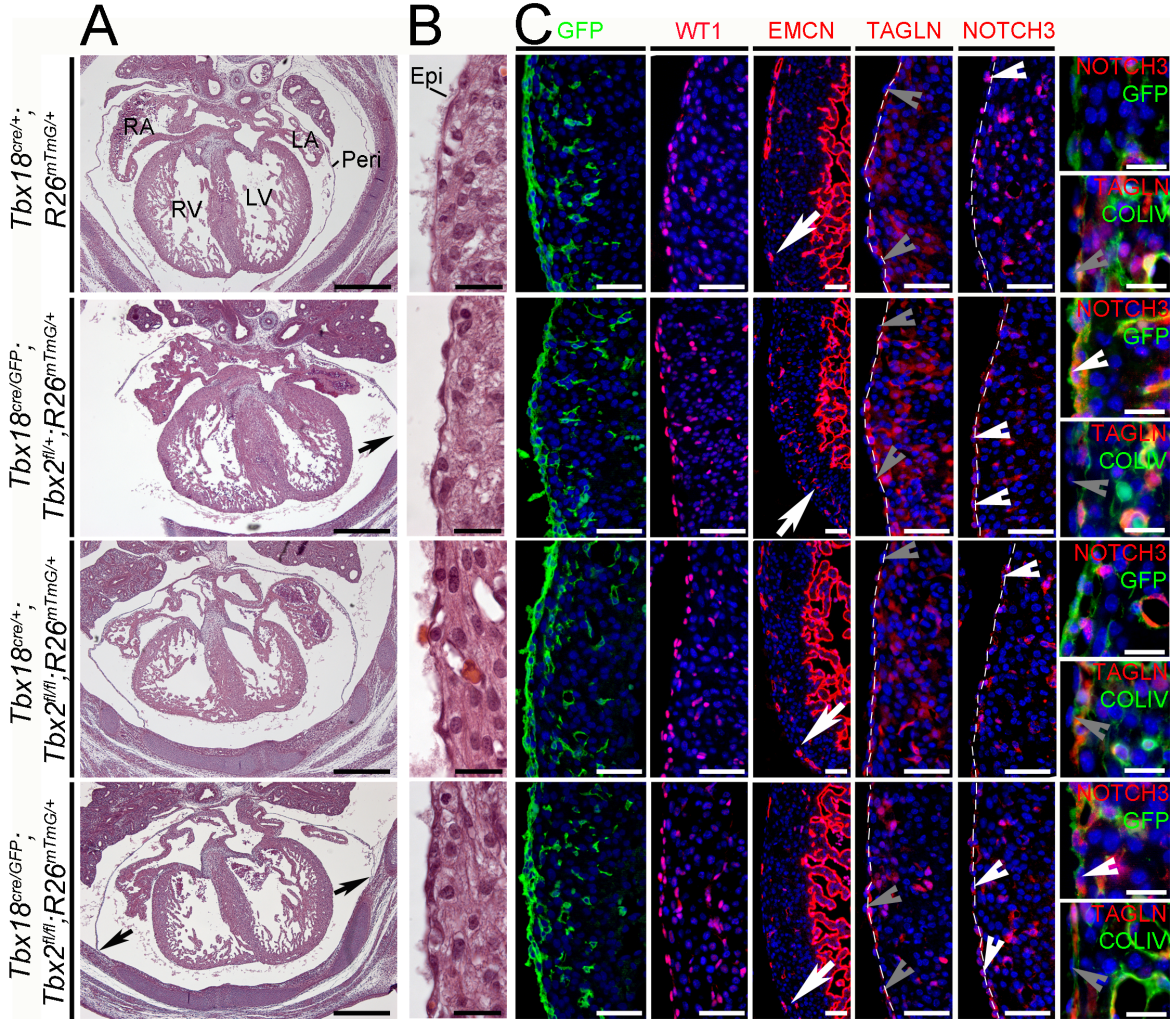
Phenotypic analysis of hearts with combined loss of *Tbx18* and *Tbx2* in the epicardium at E14.5. (A) Histological analysis of one to two embryos per genotype by hematoxylin and eosin staining of transverse heart sections does not reveal any gross morphological defects in *Tbx18*^*cre/GFP*^*;Tbx2*^*fl/fl*^ double mutant hearts compared to control (*Tbx18*^*cre/+*^) or compound mutant (*Tbx18*^*cre/GFP*^*;Tbx2*^*fl/+*^ or *Tbx18*^*cre/+*^*;Tbx2*^*fl/fl*^) embryos. The scale bars are 500 μm. The black arrows point to pericardial defects observed in *Tbx18*^*cre/GFP*^*;Tbx2*^*fl/fl*^ and *Tbx18*^*cre/GFP*^*;Tbx2*^*fl/+*^ hearts. (B) Higher magnification of the right ventricle shows a tightly attached epicardium on top of the heart in all genotypes. The scale bars are 20 μm (n = 1). (C) Immunofluorescence analysis of GFP and WT1 expression confirms epicardial integrity and subepicardial as well as myocardial localization of epicardium-derived cells in all genotypes. The scale bars represent 50 μm. The in-growing vasculature, visualized by EMCN immunofluorescence, has almost reached the apex of the right ventricle (white arrows). TAGLN is not expressed in the epicardium as emphasized by double immunofluorescence with COLIV (grey arrowheads). In contrast, NOTCH3 expression is found in the epicardium of *Tbx18*^*cre/GFP*^*;Tbx2*^*fl/fl*^ and *Tbx18*^*cre/GFP*^*;Tbx2*^*fl/+*^ mice and occasionally in *Tbx18*^*cre/+*^*;Tbx2*^*fl/fl*^ and control hearts (white arrowheads). Double staining with the *Tbx18*-lineage marker GFP indicates NOTCH3-positive cells in the epicardium of *Tbx18*^*cre/GFP*^*;Tbx2*^*fl/fl*^ and *Tbx18*^*cre/GFP*^*;Tbx2*^*fl/+*^ mice. Dashed lines mark the border between epicardium and myocardium. Scale bars in NOTCH3 and TAGLN single staining are 50 μm, and 20 μm in the double staining of these markers with GFP or COLIV. Two specimens per genotype and stage were analyzed by immunostaining. Epi, epicardium; LA, left atrium; LV, left ventricle; Peri, pericardium; RA, right atrium; RV, right ventricle.

We focused our analysis on E14.5 embryos to be able to compare our findings with that of a previous study on mice with epicardial overexpression of an activator version of TBX18 [35]. Hematoxylin and eosin staining of transverse sections through the heart region did not reveal any difference in the histological appearance of the cardiac chambers in compound mutants (*Tbx18*^*cre/GFP*^*;Tbx2*^*fl/+*^ and *Tbx18*^*cre/+*^*;Tbx2*^*fl/fl*^), and double mutants (*Tbx18*^*cre/GFP*^*;Tbx2*^*fl/fl*^) compared to control embryos (*Tbx18*^*cre/+*^); septa and valves were formed normally and the ventricular myocardium was of normal thickness. In *Tbx18*-deficient mice pleuropericardial membranes were not completely resolved from the body wall but remained laterally attached in agreement with our previous report on the role of *Tbx18* in the development of this tissue [48] (Fig 3A, arrows). Higher magnification of the histologically stained right ventricle demonstrated that the epicardium was correctly attached in double and compound mutant embryos (Fig 3B). To visualize the epicardium and its descendants on a cellular level, we analyzed GFP expression from the *R26*^*mTmG*^ reporter allele in the different mutant combinations. GFP-positive cells localized to the subepicardial space and intermingled with cardiomyocytes in double and compound mutants as in control mice; and visual inspection of transverse sections did not reveal gross changes in the number of immigrating cells (Fig 3C). Similarly, we did not detect differences in the expression of Wilms tumor 1 (WT1), a marker for epicardial and epicardium-derived cells, and more weakly for endothelial cells [45, 49] in double and compound mutant embryos compared to the control (Fig 3C). Expression of endomucin (EMCN), a marker of venous and capillary endothelial cells and of the endocardium [50], was indistinguishable in double and compound mutant and control hearts indicating that formation of the coronary plexus occurred normally (Fig 3C). We conclude that epicardial signaling is unaffected by loss of *Tbx18* and/or *Tbx2*. To clarify whether loss of *Tbx18* and *Tbx2* impairs SMC differentiation, expression of SMC proteins NOTCH3 and Transgelin (TAGLN) was analyzed. Epicardial cells were delineated by costaining for collagen IV (COLIV) expression in the basal lamina [9]. TAGLN protein expression was found in subepicardial cells and cardiomyocytes but not in epicardial cells in any of the analyzed genotypes at this stage (Fig 3C). NOTCH3 protein was detectable in up to 70% of epicardial cells of *Tbx18*^*cre/GFP*^*;Tbx2*^*fl/fl*^ and *Tbx18*^*cre/GFP*^*;Tbx2*^*fl/*+^ hearts but only in 50% of control or *Tbx18*^*cre/+*^*;Tbx2*^*fl/fl*^ epicardial cells indicating that the loss of *Tbx18* accounts for this effect (Fig 3C, S2 Fig).

**Fig 4:**
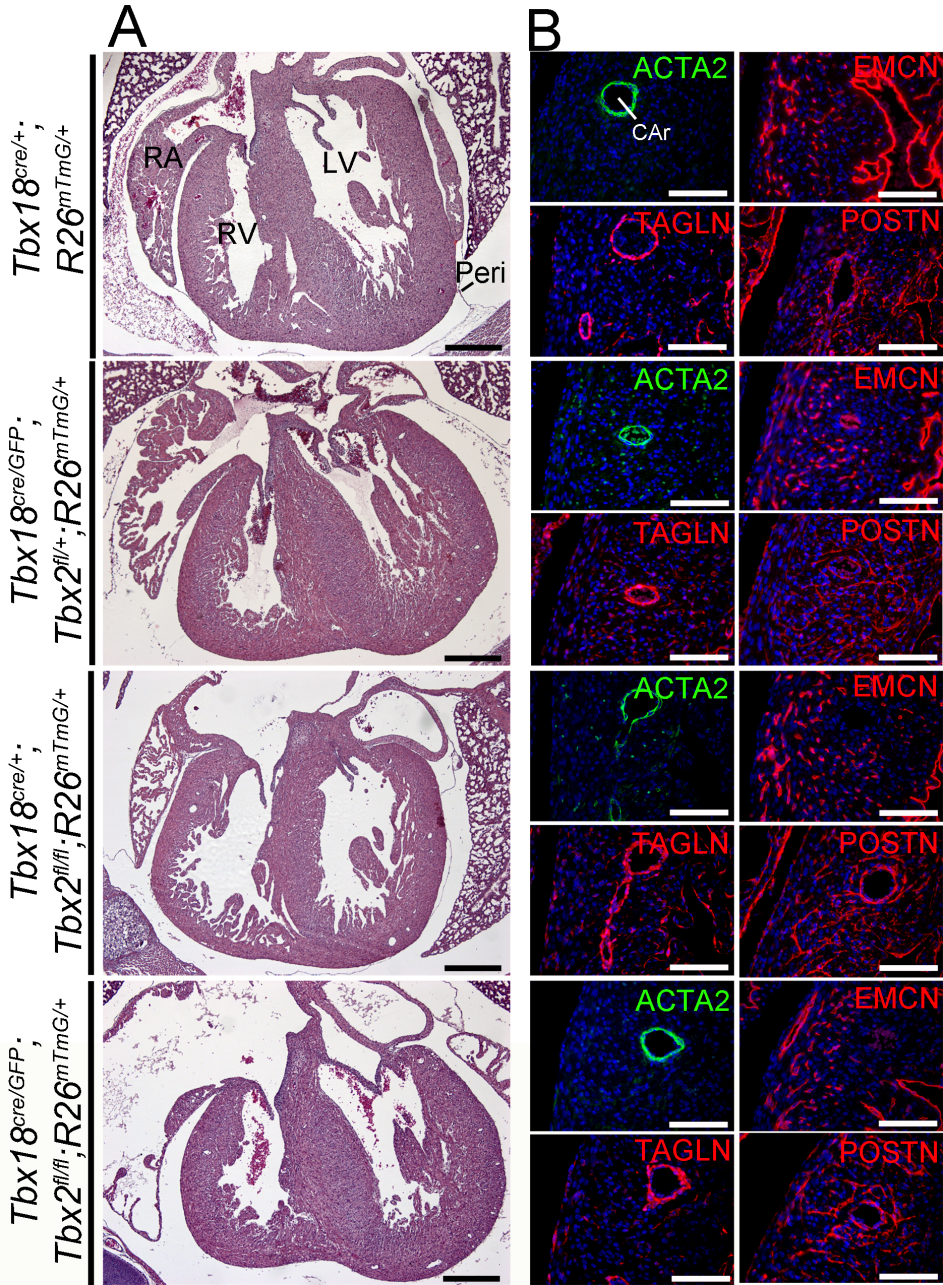
Phenotypic analysis of hearts with combined loss of T*bx18* and *Tbx2* in the epicardium at E18.5. (A) Histological analysis by hematoxylin and eosin staining of transverse sections of *Tbx18*^*cre/GFP*^*;Tbx2*^*fl/fl*^ hearts reveals a dilatation of the atria in comparison to *Tbx18*^*cre/GFP*^*;Tbx2*^*fl/+*^ or *Tbx18*^*cre/+*^*;Tbx2*^*fl/fl*^ or control (*Tbx18*^*cre/+*^) hearts. Atrial dilatation is occasionally seen in *Tbx18*-deficient mice as well (not shown). The ventricular compartment of *Tbx18*^*cre/GFP*^*;Tbx2*^*fl/fl*^ hearts appears indistinguishable from control hearts (n = 2). The scale bars are 500 μm. (B) Immunofluorescence analysis of ACTA2 and TAGLN expression shows normal differentiation of coronary SMCs and their localization to coronary arteries (CAr). Capillary density, although not quantified, appears unaffected by the loss of *Tbx18*, *Tbx2* or both genes in the epicardium as visualized by immunofluorescence against EMCN. The presence of POSTN in the myocardium confirms the formation of cardiac fibroblasts from epicardial cells in all mutants. Two specimens per genotype were analyzed. Scale bars are 100 μm. Epi, epicardium; LA, left atrium; LV, left ventricle; Peri, pericardium; RA, right atrium; RV, right ventricle.

At E18.5, epicardium-derived cells have fully differentiated into fibroblasts and SMCs, constituting the cardiac fibrous skeleton and completing the formation of the coronary vasculature. Hematoxylin and eosin staining of transverse sections through the heart at this stage did not reveal histological defects in addition to the pericardial changes observed in *Tbx18*-deficient embryos at E14.5 (Fig 4A). Coronary arteries developed normally in *Tbx18/Tbx2* compound and double mutant hearts and were surrounded by SMCs expressing actin, alpha 2, smooth muscle, aorta (ACTA2) and TAGLN (Fig 4B) and the late SMC differentiation marker myosin, heavy polypeptide 11, smooth muscle (MYH11) (S3 Fig). EMCN expression in mutants was indistinguishable from the control indicating the formation of a normal endothelial network in the coronary vasculature. Finally, the formation and distribution of periostin (POSTN)-positive fibroblasts in the myocardium was not affected by the individual or combined loss of *Tbx2* and *Tbx18* (Fig 4B). We conclude, that *Tbx2* and *Tbx18* are neither individually nor combinatorially required in the epicardium.

### Misexpression of human *TBX2* in epicardial cells and their progeny does not affect cardiac development

**Fig 5:**
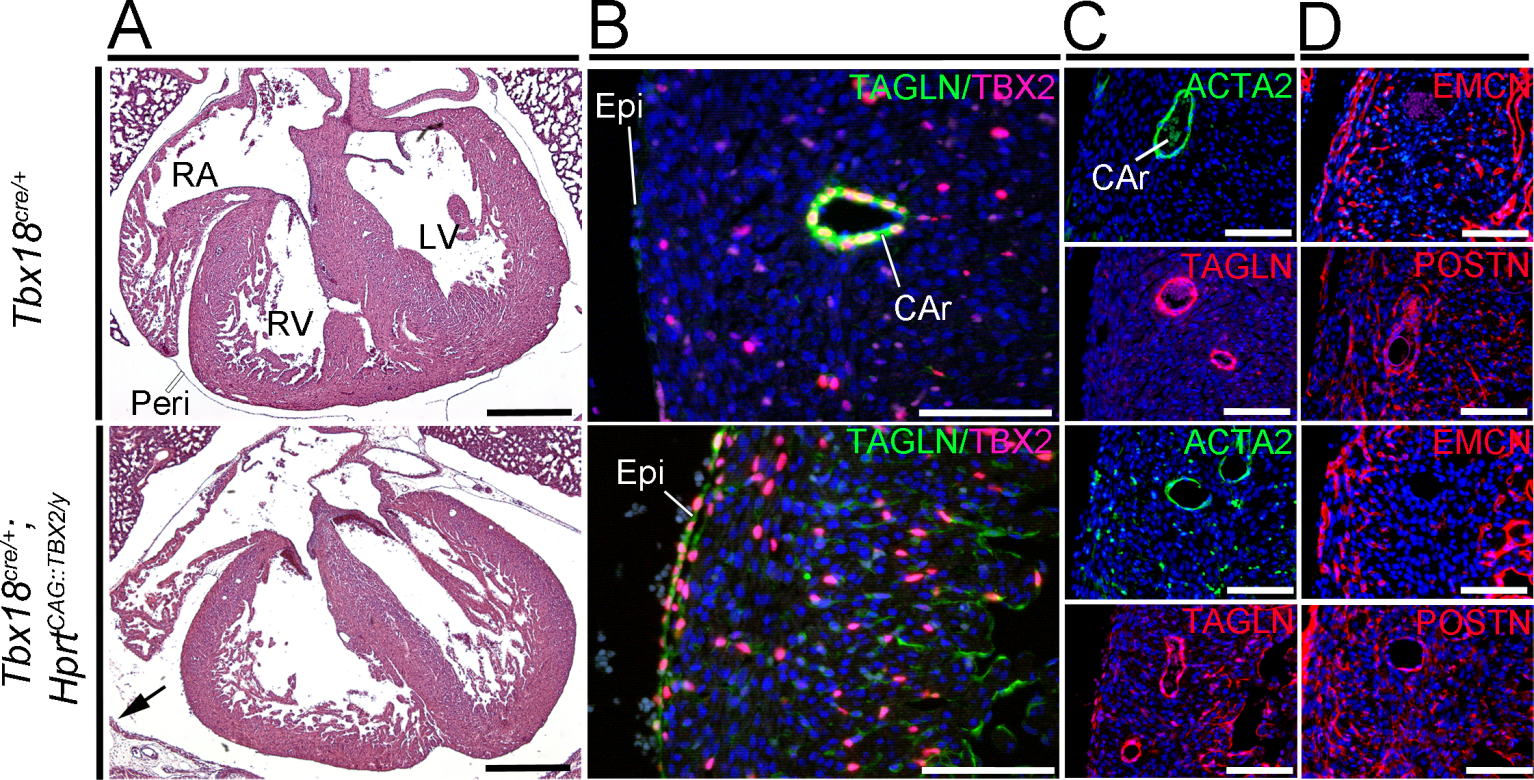
Phenotypic analysis of E18.5 hearts with epicardial misexpression of human *TBX2*. (A) Histological analysis (n = 2) by hematoxylin and eosin staining of transverse sections of *Tbx18*^*cre/+*^*;Hprt*^*CAG*::*TBX2/y*^ hearts reveals pericardial defects (black arrow) but no further anomalies compared to the control (*Tbx18*^*cre/+*^). Scale bars are 500 μm. (B-D) Immunofluorescence analysis (n = 4) of indicated proteins on transverse sections of *Tbx18*^*cre/+*^ (control) and *Tbx18*^*cre/+*^*;Hprt*^*CAG*::*TBX2/y*^ hearts. Shown are magnified regions of the right ventricle. Scale bars are 100 μm. (B) Human TBX2 protein and TAGLN is found in the epicardium and in epicardium-derived cells in *Tbx18*^*cre/+*^*;Hprt*^*CAG*::*TBX2/y*^ hearts. Note that the level of human TBX2 expression is similar to the level of endogenous mouse TBX2 protein. (C) Coronary vessels are surrounded by ACTA2- and TAGLN-expressing SMCs in *Tbx18*^*cre/+*^*;Hprt*^*CAG*::*TBX2/y*^ hearts at E18.5, but additional ACTA2- and TAGLN-positive cells are detected in the myocardium of these hearts. (D) Small EMCN-positive coronary vessels form in comparable densities in *Tbx18*^*cre/+*^*;Hprt*^*CAG*::*TBX2/y*^ and control hearts. Intramyocardial deposition of POSTN was detected in both genotypes in a comparable fashion. CAr, coronary artery; Epi, epicardium; LV, left ventricle; Peri, pericardium; RA, right atrium; RV, right ventricle.

Although the loss of *Tbx2* does not effect epicardial development, its localized expression in a subset of epicardial cells may indicate an involvement of the gene in the specification of distinct epicardial sublineages. To study the potential effect of *Tbx2* on lineage segregation in the epicardium, we generated mice ectopically expressing human *TBX2* in the whole epicardium and its descendants. For this purpose *Tbx18*^*cre/+*^*;R26*^*mTmG/mTmG*^ males were mated to females homozygous for an integration of a cre-inducible *TBX2* expression cassette in the X-chromosomal *Hprt* locus (*Hprt*^*CAG*::*TBX2/CAG*::*TBX2*^) [26]. Since female *Tbx18*^*cre/+*^*;R26*^*mTmG/+*^*;Hprt*^*CAG*::*TBX2/+*^ embryos express the transgene in a mosaic fashion due to X-chromosome inactivation, we subsequently only analyzed male *Tbx18*^*cre/+*^*;R26*^*mTmG/+*^*;Hprt*^*CAG*::*TBX2/y*^ embryos that express the transgene homogenously. *Tbx18*^*cre/+*^*;R26*^*mTmG/+*^*;Hprt*^*CAG*::*TBX2/y*^ embryos were present in the expected Mendelian ratio at E18.5 (n = 10/42). Histological analysis revealed that the pleuropericardial membranes were not fully detached from the body wall. However, septa and valves were unaffected and the ventricular walls exhibited normal thickness and trabeculation (Fig 5A). Immunofluorescence analysis confirmed expression of human TBX2 protein in the epicardium and epicardium-derived cells at levels similar to that of endogenous mouse TBX2 in coronary SMCs. Weak ectopic expression of TAGLN was associated with human TBX2 protein in the epicardium and the myocardium in *Tbx18*^*cre/+*^*;R26*^*mTmG/+*^*;Hprt*^*CAG*::*TBX2/y*^ mice (Fig 5B). Formation of coronary arteries was unaffected although the surrounding SMC layer appeared thinned in *Tbx18*^*cre/+*^*;R26*^*mTmG/+*^*;Hprt*^*CAG*::*TBX2/y*^ hearts as indicated by staining for the SM proteins ACTA2 and TAGLN (Fig 5C). However, the contribution of epicardium-derived cells to the SM lineage surrounding coronary arteries was unaffected in *Tbx18*^*cre/+*^*;R26*^*mTmG/+*^*;Hprt*^*CAG*::*TBX2/y*^ hearts (S4 Fig). In contrast to epicardium-derived cells in control hearts, TAGLN but not ACTA2 was ectopically expressed in epicardial cells and in a majority of epicardium-derived cells of *Tbx18*^*cre/+*^*;R26*^*mTmG/+*^*;Hprt*^*CAG*::*TBX2/y*^ hearts at E18.5 (Fig 5A and 5B, S4 Fig: red arrow heads). EMCN expression in mutants was unchanged from the control indicating the formation of a normal venous and capillary network in the coronary vasculature. Finally, the formation and distribution of POSTN-positive fibroblasts in the myocardium was normal in *Tbx18*^*cre/+*^*;R26*^*mTmG/+*^*;Hprt*^*CAG*::*TBX2/y*^ hearts at this stage (Fig 5D, S4 Fig). Together, this suggests that homogenous epicardial misexpression of human TBX2 does not impair formation, migration and differentiation of the epicardial and epicardium-derived cells. Although ectopic expression of TAGLN in epicardial and epicardium-derived cells of *Tbx18*^*cre/+*^*;R26*^*mTmG/+*^*;Hprt*^*CAG*::*TBX2/y*^ hearts was observed, the differentiation potential of those cells was maintained.

### Reduction of the *Tbx20* gene dosage in combination with the loss of *Tbx18* does not affect epicardial EMT

TBX20 like TBX18 is a member of the TBX1 subfamily, and can act either as a transcriptional repressor [22, 51] or as a transcriptional activator [23, 52]. It is therefore possible that a TBX20 repressor function acts redundantly with TBX18 in epicardial development. As *Tbx20*-deficient mice die shortly after E9.5 [20], and a floxed allele of *Tbx20* was not available to us, we decided to analyze mice compound mutant for a null allele of *Tbx18* (*Tbx18*^*cre*^ *and Tbx18*^*LacZ*^) [38] and a null allele of *Tbx20* (*Tbx20*^*LacZ*^) that we previously generated and characterized [20], to determine the effect of a reduced *Tbx20* gene dosage in a *Tbx18*-deficient background. Notably, *Tbx18*^*cre/cre*^*;Tbx20*^*LacZ/+*^ mutant embryos were underrepresented in E14.5 litters derived from matings of *Tbx18*^*cre/+*^*;Tbx20*^*LacZ/+*^*;R26*^*mTmG/+*^ male with *Tbx18*^*cre/+*^*;Tbx20*^*LacZ/+*^*;R26*^*mTmG/+*^ female mice (expected 16.7%, obtained 4%, n = 2/50) indicating an early lethality of these compound mutants. All other *Tbx20*^*LacZ/+*^ compound mutants (*Tbx18*^*cre/+*^*;Tbx20*^*LacZ/+*^: n = 13/50, *Tbx18*^*+/+*^*;Tbx20*^*LacZ/+*^: n = 4/50) were slightly underrepresented as well whereas *Tbx18*^*cre/LacZ*^*;Tbx20*^*+/+*^ (n = 13/50) mutants and *Tbx18*^*cre/+*^*;Tbx20*^*+/+*^ mutants (n = 14/50) were overrepresented.

**Fig 6:**
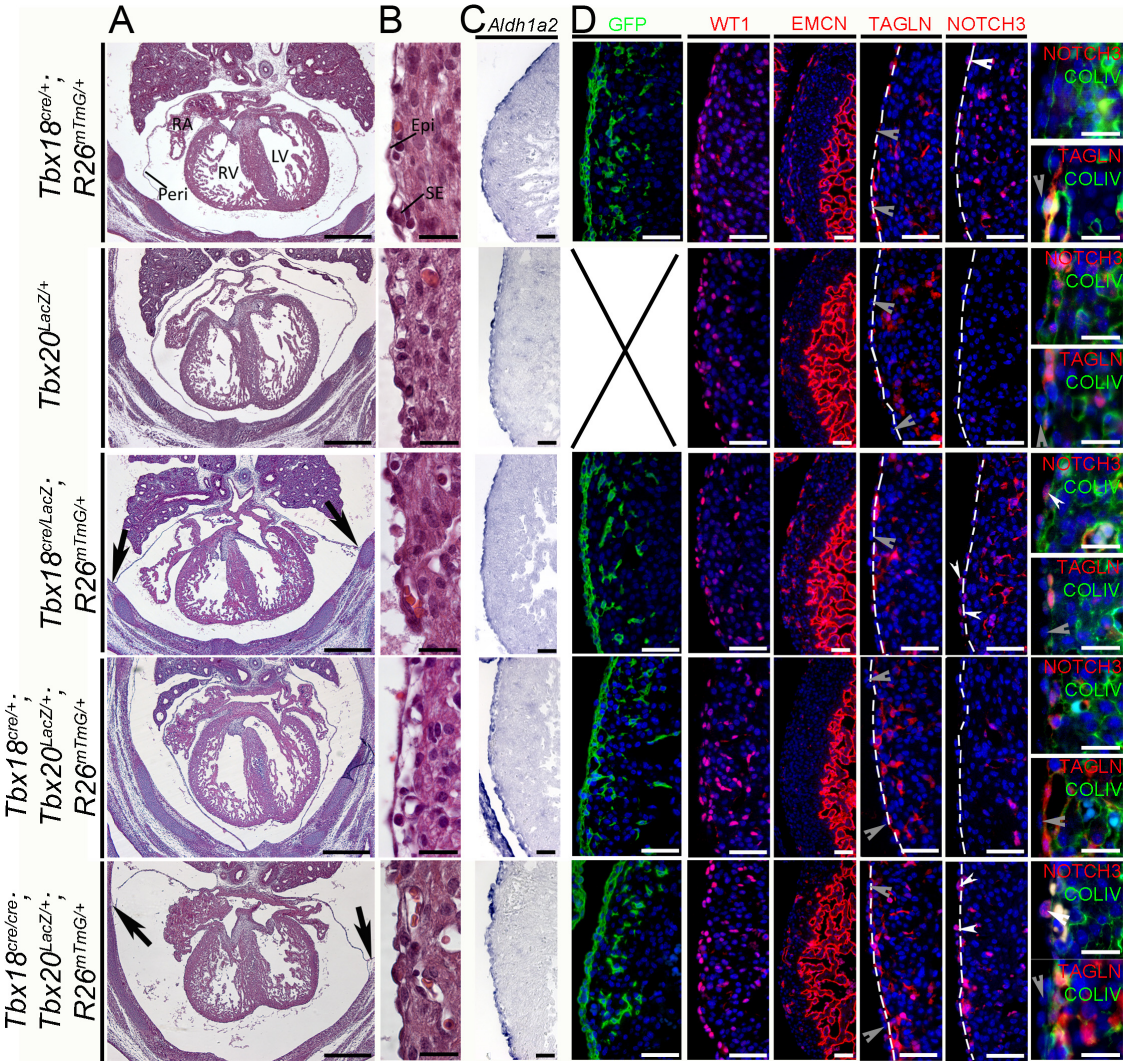
Phenotypic analysis of E14.5 hearts with combined loss of *Tbx18* and one *Tbx20* allele. (A, B) Histological analysis by hematoxylin and eosin staining of transverse sections of E14.5 hearts shows that the reduction of the *Tbx20* gene dosage in a *Tbx18*-mutant background does not affect epicardial and myocardial integrity. Pericardial defects are observed in *Tbx18*^*cre/cre*^*;Tbx20*^*LacZ/+*^ as well as in *Tbx18*^*cre/LacZ*^ mice (black arrows in A). The scale bars are 500 μm. (B) The right ventricular epicardium (Epi) shows a cellular monolayer on top of a less dense subepicardial layer (SE) and the myocardium in all genotypes. The scale bars are 20 μm. (C) Section *in situ* hybridization against *Aldh1a2* confirms epicardial integrity. The scale bars are 50 μm. (D) Epicardial cells, immunologically stained for the *Tbx18*-lineage label GFP (from an introduced *Rosa26*^*mTmG*^ allele) or the epicardial marker WT1, undergo EMT and populate the subepicardial space as well as the myocardium in a similar fashion in all mutant and control mice. The coronary plexus forms normally in *Tbx18*^*cre/cre*^*;Tbx20*^*LacZ/+*^ mice and reaches the right ventricular apex similar to *Tbx18*^*cre/LacZ*^ single mutant and control mice as indicated by immunofluorescence against EMCN. The scale bars are 50 μm. A premature differentiation of epicardial cells into SMCs does not occur in any mutant as visualized by immunofluorescence against TAGLN. Grey arrowheads indicate TAGLN-negative epicardial cells. TAGLN-expressing cells are found within the myocardium and subepicardial space as confirmed by double staining with COLIV. Immunofluorescence against NOTCH3 on the other hand demonstrates the presence of few NOTCH3-positive cells in the epicardium of *Tbx18*^*cre/+*^ controls and an increased number of NOTCH3-positive epicardial cells in *Tbx18*^*cre/LacZ*^ and *Tbx18*^*cre/cre*^*;Tbx20*^*LacZ/+*^ mutants whereas none are observed in the depicted sections of *Tbx20*^*LacZ/+*^ or *Tbx18*^*cre/+*^*;Tbx20*^*LacZ/+*^ mutants. These results are confirmed by double immunofluorescence against NOTCH3 and COLIV. White arrowheads point toward NOTCH3-positive cells within the epicardium. Scale bars are 50 μm for TAGLN and NOTCH3 staining and 20 μm for double staining with COLIV. Dashed lines indicate the epicardial-myocardial border. Two specimens per genotype were analyzed. Epi, epicardium; LA, left atrium; LV, left ventricle; Peri, pericardium; RA, right atrium; RV, right ventricle.

Histological stainings of transverse sections of *Tbx18*^*cre/cre*^*;Tbx20*^*LacZ/+*^ hearts revealed normal chamber architecture and septa formation but pericardial defects similar to *Tbx18-null* mice (black arrows in Fig 6A). Higher magnification of the right ventricular epicardium revealed a monolayer of flattened cells covering the myocardium in all mutants; a thin subepicardial space was formed in *Tbx18*^*cre/cre*^*;Tbx20*^*LacZ/+*^ hearts as in *Tbx18*^*cre/LacZ*^ and control hearts (Fig 6B). Blood filled vessels visualized by the presence of eosin-positive red blood cells grew into the subepicardial space in all mutants. Section *in situ* hybridization of transverse sections against *Aldh1a2* confirmed the structural integrity of the epicardium (Fig 6C), and immunofluorescence of EMCN expression demonstrated that the coronary plexus was recruited correctly (Fig 6D). Both findings indicate that the epicardial-myocardial crosstalk is undisturbed. Immunofluorescence of the lineage label GFP and the epicardial marker WT1 further proved the formation of a complete epicardium that was tightly attached to the surface of the heart (Fig 6D). Epicardium-derived cells were present in the subepicardium and myocardium in mutants as in controls as visualized by expression of GFP and/or WT1. The amount of GFP- and WT1-positive cells entering the myocardium and their distribution between subepicardial and myocardial compartments was unaltered indicating proper EMT and immigration patterns in *Tbx18*^*cre/cre*^*;Tbx20*^*LacZ/+*^, *Tbx18*^*cre/LacZ*^, *Tbx18*^*cre/+*^*;Tbx20*^*LacZ/+*^ and control hearts (Fig 6D). Expression of TAGLN was restricted to the subepicardial space in *Tbx18*^*cre/cre*^*;Tbx20*^*LacZ/+*^ hearts (Fig 6D) arguing against premature SMC differentiation of epicardial cells. Expression of NOTCH3 was observed in some epicardial cells of *Tbx18-null* mutant embryos but was not enhanced by the additional loss of *Tbx20* (S2 Fig). Hence, the loss of one *Tbx20* allele does not alter epicardial development in wild-type or *Tbx18*-deficient mice.

## Discussion

Here, we found that *Tbx2*, *Tbx20* and *Tbx5* are co-expressed with *Tbx18* at different stages of epicardial development. Our genetic experiments showed that *Tbx2* is dispensable for epicardial development, and that neither *Tbx2* nor *Tbx20* redundantly interact with *Tbx18* in any of the subprograms important for formation, migration and differentiation of epicardial cells in mouse embryos.

### *Tbx18* and combinatorial interaction with *Tbx* genes in epicardial development

Our previous analysis demonstrated that loss of *Tbx18* does not affect epicardial function whereas epicardial-specific misexpression of an activator version of TBX18 (TBX18VP16) led to premature SMC differentiation of epicardial cells [35]. This suggested that TBX18 possibly together with a related transcriptional repressor maintains the precursor character of epicardial cells by repressing SMC differentiation. Our sensitive PCR and *in situ* hybridization methods detected expression of three additional cardiac *Tbx* genes, *Tbx5*, *Tbx20* and *Tbx2* in the proepicardium and epicardium. Expression of *Tbx5* was detected in the E9.5 proepicardium, and in the E10.5–11.5 epicardium but was subsequently down-regulated. A recent report found a similar pattern of expression both on the level of *Tbx5* mRNA and TBX5 protein, and uncovered that epicardial-specific loss of *Tbx5* is associated with blebbing and reduced EMT but not with premature SMC differentiation of epicardial cells. Augmented expression of Tbx5 in the proepicardium led to reduced proepicardial migration and enhanced apoptosis in chick embryos [30]. Together, this suggests that precise levels of *Tbx5* are important for formation and migration of proepicardial cells. To date, TBX5 has only been characterized as a transcriptional activator in cardiac development [30, 53] whereas a Groucho-dependent role as a transcriptional repressor was assigned to TBX18 [36]. Although it cannot be formally excluded that both factors behave in a biochemically equivalent fashion (as activators or repressors) in epicardial development, the phenotypic differences between *Tbx5*- and *Tbx18*-deficient embryos argue against a redundant function. Given our earlier finding that TBX18 can compete with TBX5 for DNA-binding sites *in vitro* [36], it cannot be excluded, however, that TBX18 is important to fine-tune the transcriptional responses to TBX5 by selectively repressing some of the TBX5 target genes. More detailed transcriptional profiling of *Tbx5*- and *Tbx18*-deficient (pro-)epicardial cells may help to test such a scenario.

We also detected expression of *Tbx20* in the proepicardium and early epicardium albeit unambiguous assignment to this tissue was hampered by strong myocardial expression of the gene. *Tbx20*-deficient embryos form a proepicardium but an analysis of subsequent epicardial development is impossible due to the lethality at E9.5 [20]. Mice heterozygous for a *Tbx20* null allele survive into adulthood with diverse cardiac pathologies, including defects of septation and valvulogenesis and cardiomyopathy that however, do not correspond to an epicardial requirement of the gene [22, 54]. Here, we have shown that mice compound mutant for *Tbx18* and *Tbx20* do not exhibit defects in epicardial development. Despite the fact that TBX20 can act as a repressor in developing and mature hearts [22, 51, 52, 55], these genetic findings argue that *Tbx20* neither on its own nor in combination with *Tbx18* plays an essential role in the development of the epicardium. Since TBX20 can also act as a transcriptional activator [55], it may cooperate with TBX5 in regulating proepicardial and early epicardial development. Such a possibility may be addressed by proepicardial-specific deletion of both *Tbx5* and *Tbx20* in the mouse.

Finally, we detected expression of *Tbx2* (but not of the closely related *Tbx3* gene) in a subset of proepicardial and epicardial cells in the developing mouse heart. Since *Tbx2* like *Tbx18* encodes a strong transcriptional repressor [36, 46], we focused our genetic studies on the role of *Tbx2* and its possible redundancy with *Tbx18* in epicardial development. However, neither embryos with an epicardial deletion of *Tbx2* nor with combined deficiency of *Tbx18* and *Tbx2* exhibited epicardial defects, strongly arguing against an individual or combined role for *Tbx2* in epicardial development. Similar to *Tbx18* [35], misexpression of *TBX2* in the epicardial lineage did not affect epicardial function nor did it prevent SMC differentiation of epicardium-derived cells. Both for TBX2 and TBX18, this may reflect the lack of cofactors necessary to exert this function. Since TBX2 (and TBX3) have been characterized as strong competitive inhibitors of TBX5-activated gene programs in the atrioventricular canal and outflow tract [26, 56], the possibility again exists, that TBX2 represses TBX5 target genes in individual proepicardial and epicardial cells.

While TBX5, TBX20 and TBX2 do not hold promise as cooperation partners of TBX18 in maintenance of epicardial integrity, complex dose-dependent antagonistic and synergistic interactions between the different TBX family members may exist that play an important role in generating a molecular heterogeneity in fate decisions in proepicardial and epicardial cells as in other developmental contexts [57]. Other T-box proteins that have not yet been associated with cardiac development might additionally be expressed in the epicardium to feed into this regulatory network.

### TBX18 function in repression of SMC differentiation may depend on additional cofactors

While the identity of a TBX protein acting redundantly with TBX18 in repression of SMC differentiation of epicardial cells remains enigmatic, the maintenance of epicardial precursor character in *Tbx18*-deficient embryos may also reflect lack of an activator of the SMC program in these cells. Interestingly, Wu and colleagues recently analyzed the potential of *Tbx18* to inhibit a SMC differentiation program *in vitro*. They used C3H10T1/2 cells, multipotent mesenchymal progenitors that initiate the SMC pathway when exposed to TGFß1, and showed that cells transfected with a *Tbx18* expression vector exhibit a markedly reduced expression of SMC markers. This result was not due to increased apoptosis or reduced proliferation indicating that TBX18 is indeed able to suppress a SMC differentiation pathway. Moreover, they found in transactivation assays that TBX18 inhibits Serum response factor (SRF)-CArG-box dependent activation of the promotors of the SMC-specific genes *Tagln*, *Fos* and *Actg2* [37]. Lack of SMC differentiation in *Tbx18*-deficient epicardial cells may therefore simply reflect the lack of expression of the master activators of the SMC program, *Myocardin* (*Myocd*) and/or *Srf* [58] in the epicardium. TBX18VP16, an activator version of TBX18, on the other hand may be able to activate target genes usually repressed by TBX18 independently of co-factors leading to premature differentiation of the epicardium into SMCs [35].

It is worth to note that NOTCH3 expression was upregulated in epicardial cells overexpressing TBX18VP16 as well as in cells of *Tbx18*-null epicardia. *Notch3* contains functional T-sites in the zebrafish [59] suggesting that *Notch3* expression is activated independently from MYOCD/SRF but is directly repressed by TBX18. Although epicardial overexpression of the intracellular fragment of NOTCH is sufficient to induce SMC differentiation [9], lack of the appropriate ligand in *Tbx18*-deficient epicardial cells will prevent activation of *Notch3* and induction of the SMC pathway.

## Supporting Information

S1 Fig Absence of TBX18 and TBX2 protein in mutant embryos. (A) Immunofluorescence analysis of *TBX18* on transverse E10.5 sections through right ventricles of control and *Tbx18*^*GFP/GFP*^ mice. Epicardial and pericardial cells express TBX18 in the control (white arrows) but not in *Tbx18*^*GFP/GFP*^ mice. (B) Immunofluorescence analysis of *TBX2* on transverse E10.5 sections through right ventricles of control and *Tbx18*^*cre/+*^*;Tbx2*^*fl/fl*^*;R26*^*mTmG/+*^ mice. Pericardial cells are positive for TBX2 in the control but not in *Tbx18*^*cre/+*^*;Tbx2*^*fl/fl*^*;R26*^*mTmG/+*^ mice. Scale bars are 50 μm. Epi, epicardium; Peri, Pericardium; RA, right atrium; RV, right ventricle.
(TIF)

S2 Fig Increase of NOTCH3^+^-epicardial cells in *Tbx18*-null mice is independent from *Tbx2* or *Tbx20*. In order to quantify NOTCH3-expressing epicardial cells, immunofluorescent stainings against NOTCH3 were analyzed. (A) Quantification of NOTCH3-positive epicardial cells in *Tbx18*^*cre/GFP*^*;Tbx2*^*fl/fl*^ (70.0±4.4%), *Tbx18*^*cre/GFP*^*;Tbx2*^*fl/+*^ (67.9±8.2%), *Tbx18*^*cre/+*^;*Tbx2*^*fl/fl*^ (52.2±13.8%) and *Tbx18*^*cre/+*^ hearts. Two specimens of each genotype were analyzed and the ratio of NOTCH3-positive cells within the right ventricular epicardium was determined and displayed as percentage. Error bars indicate the standard deviation. (B) Ratio of NOTCH3-expressing cells within the epicardium of *Tbx18*^*cre/cre*^*;Tbx20*^*LacZ/+*^ (74.3±10.4%, n = 3), *Tbx18*^*cre/LacZ*^ (82.9±9.0%, n = 5), *Tbx18*^*cre/+*^*;Tbx20*^*LacZ/+*^ (61.7±5.3%, n = 2) mutant hearts and *Tbx18*^*cre/+*^ (51.2±14.2%, n = 6) as well as *Tbx20*^*LacZ/+*^ (55.0±11.8%, n = 2) control hearts is displayed as percentage. Number of specimens per genotype as indicated and error bars represent the standard deviation. (C) Direct comparison of the ratio of NOTCH3-positive cells in *Tbx18*^*cre/LacZ*^ (82.9±9.0%) and *Tbx18*^*cre/+*^ (54.5±13.0%) mutants. Five specimens per genotype were analyzed and the standard deviation blotted as error bar. Student’s t-test confirmed the significance of these results.
(TIF)

S3 Fig *Myh11* expression in coronary arteries is unchanged in *Tbx18*- and *Tbx2*-deficient hearts at E18.5. (A) *In situ* hybridization analysis of *Myh11* expression on transverse sections of hearts of control, *Tbx18*^*GFP/GFP*^ and *Tbx18*^*cre/+*^*;Tbx2*^*fl/fl*^*;R26*^*mTmG/+*^ mice at E18.5. As in control hearts, SMCs of the coronary arteries of both mutant hearts show *Myh11* expression. (B) Shown are higher magnifications of the boxed areas in the right ventricle. Scale bars are as shown. CAr, coronary artery; LV, left ventricle; Peri, pericardium; RA, right atrium; RV, right ventricle.
(TIF)

S4 Fig Normal fate of epicardium-derived cells in *Tbx18*^*cre/+*^*;R26*^*mTmG/+*^*;Hprt*^*CAG*::*TBX2/y*^ mice. Epicardial cells stained for the lineage label GFP enter the myocardium of *Tbx18*^*cre/+*^*;R26*^*mTmG/+*^*;Hprt*^*CAG*::*TBX2/y*^ hearts as in *Tbx18*^*cre/+*^*;R26*^*mTmG/+*^*;Hprt*^*+/+*^ controls and contribute to coronary SMCs as indicated by double-immunofluorescence staining against the SMC marker proteins ACTA2 or TAGLN and GFP. Besides, intermyocardial epicardium-derived cells express TAGLN but not ACTA2 in *Tbx18*^*cre/+*^*;R26*^*mTmG/+*^*;Hprt*^*CAG*::*TBX2/y*^ hearts which was not observed in control hearts at E18.5 (red arrowheads). As in control hearts, epicardium-derived cells of mutant hearts contribute partially to cardiac fibroblasts as indicated by double staining for the epicardial lineage marker GFP and the fibroblast marker protein POSTN. The arrows point towards interstitial epicardium-derived fibroblasts whereas the arrowheads mark coronary fibroblasts derived from the epicardium. The number of analyzed specimen is two and the error bar represents 40 μm. CAr, coronary artery.
(TIF)

S1 Table RT-PCR primer and conditions. The listed RT-PCR primer and conditions were used for qualitative RT-PCR experiments.
(XLSX)
