# Supplementary figures and images for: Lack of Genetic Interaction between Tbx18 and Tbx2/Tbx20 in Mouse Epicardial Development
Source: PLoS One. 2016 Jun 2;11(6):e0156787. doi: 10.1371/journal.pone.0156787 (PMC4890940; doi:10.1371/journal.pone.0156787)

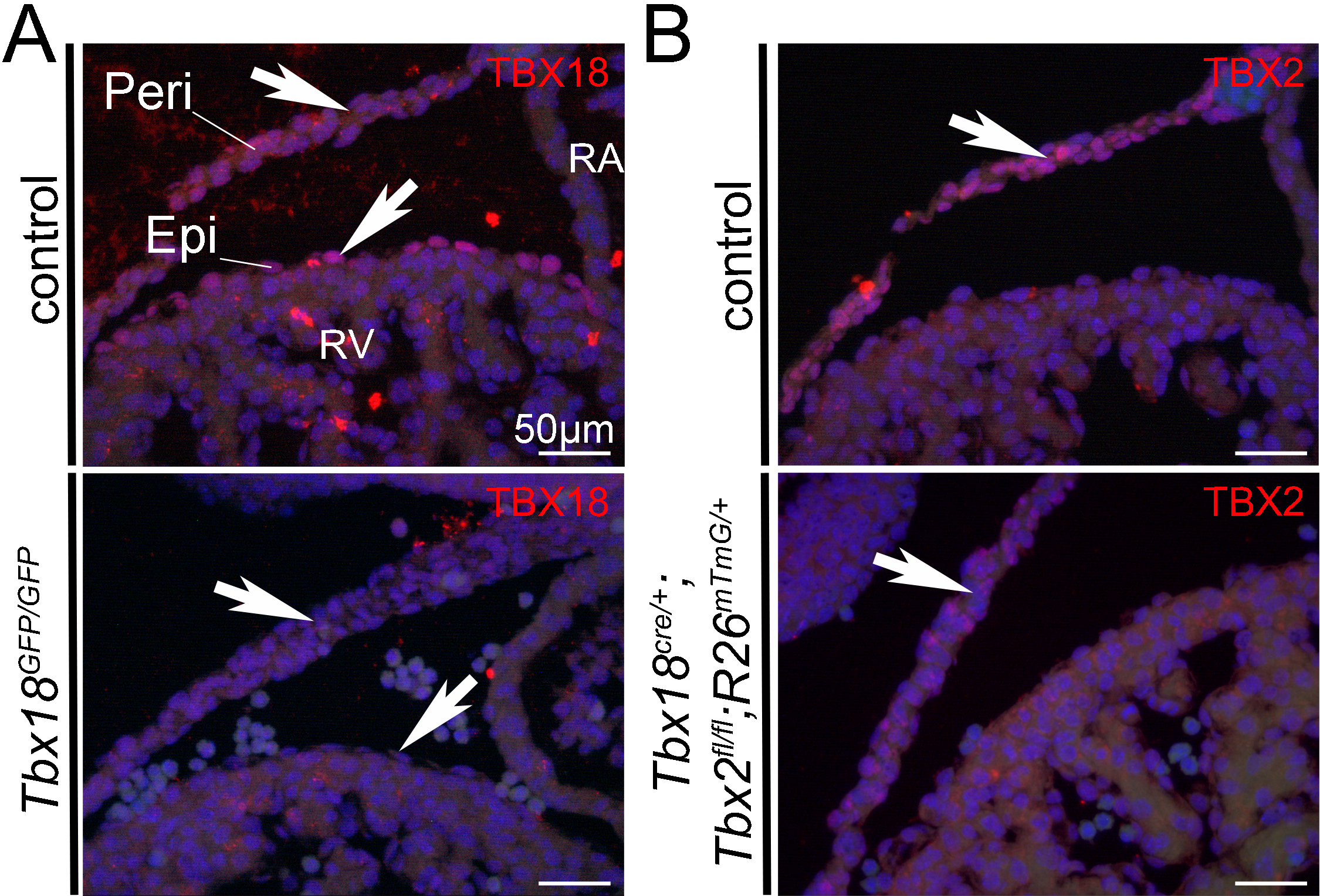

Supplement: S1 Fig — (A) Immunofluorescence analysis of TBX18 on transverse E10.5 sections through right ventricles of control and Tbx18GFP/GFP mice. Epicardial and pericardial cells express TBX18 in the control (white arrows) but not in Tbx18GFP/GFP mice. (B) Immunofluorescence analysis of TBX2 on transverse E10.5 sections through right ventricles of control and Tbx18cre/+;Tbx2fl/fl;R26mTmG/+ mice. Pericardial cells are positive for TBX2 in the control but not in Tbx18cre/+;Tbx2fl/fl;R26mTmG/+ mice. Scale bars are 50 μm. Epi, epicardium; Peri, Pericardium; RA, right atrium; RV, right ventricle. (TIF) [file pone.0156787.s001.tif]

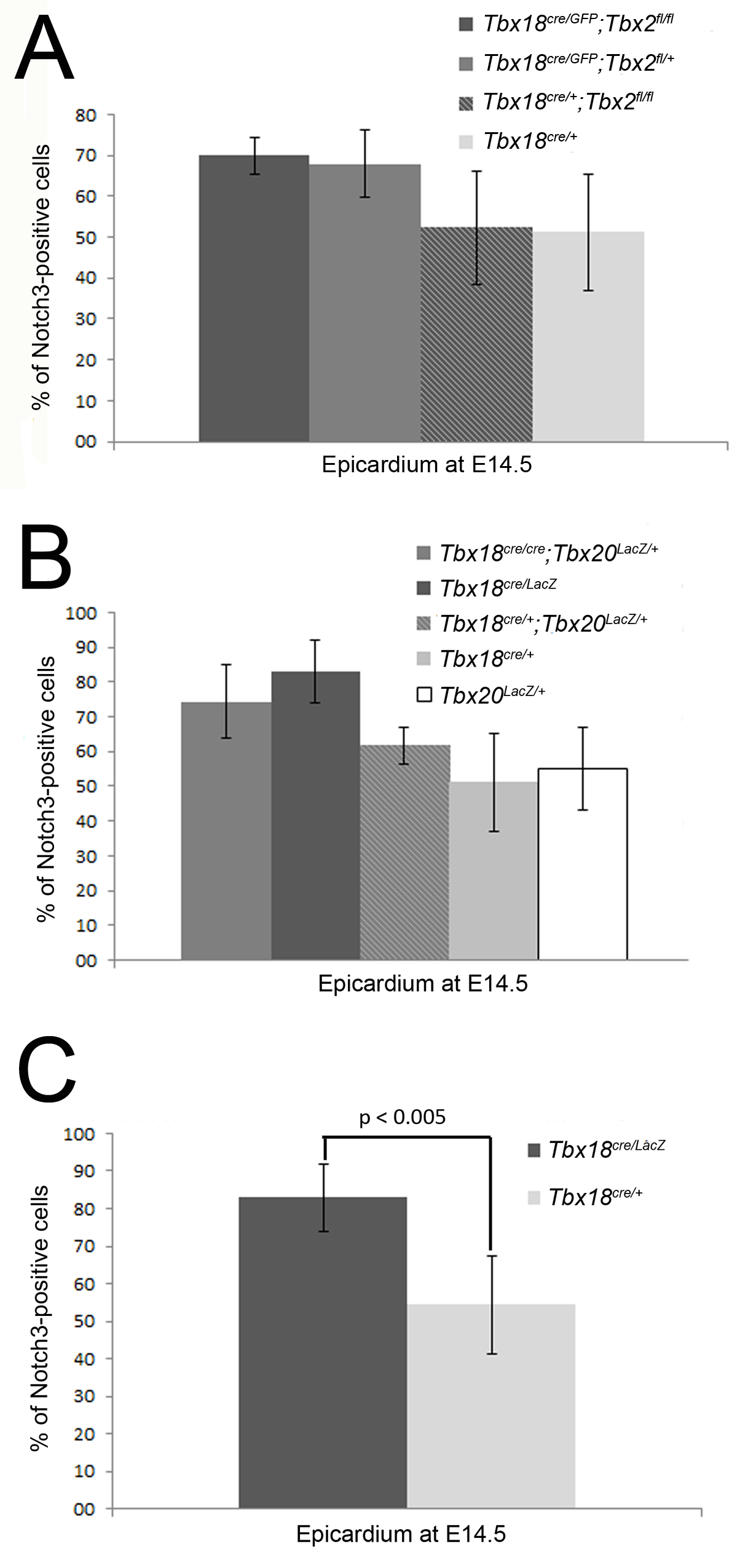

Supplement: S2 Fig — In order to quantify NOTCH3-expressing epicardial cells, immunofluorescent stainings against NOTCH3 were analyzed. (A) Quantification of NOTCH3-positive epicardial cells in Tbx18cre/GFP;Tbx2fl/fl (70.0±4.4%), Tbx18cre/GFP;Tbx2fl/+ (67.9±8.2%), Tbx18cre/+;Tbx2fl/fl (52.2±13.8%) and Tbx18cre/+ hearts. Two specimens of each genotype were analyzed and the ratio of NOTCH3-positive cells within the right ventricular epicardium was determined and displayed as percentage. Error bars indicate the standard deviation. (B) Ratio of NOTCH3-expressing cells within the epicardium of Tbx18cre/cre;Tbx20LacZ/+ (74.3±10.4%, n = 3), Tbx18cre/LacZ (82.9±9.0%, n = 5), Tbx18cre/+;Tbx20LacZ/+ (61.7±5.3%, n = 2) mutant hearts and Tbx18cre/+ (51.2±14.2%, n = 6) as well as Tbx20LacZ/+ (55.0±11.8%, n = 2) control hearts is displayed as percentage. Number of specimens per genotype as indicated and error bars represent the standard deviation. (C) Direct comparison of the ratio of NOTCH3-positive cells in Tbx18cre/LacZ (82.9±9.0%) and Tbx18cre/+ (54.5±13.0%) mutants. Five specimens per genotype were analyzed and the standard deviation blotted as error bar. Student’s t-test confirmed the significance of these results. (TIF) [file pone.0156787.s002.tif]

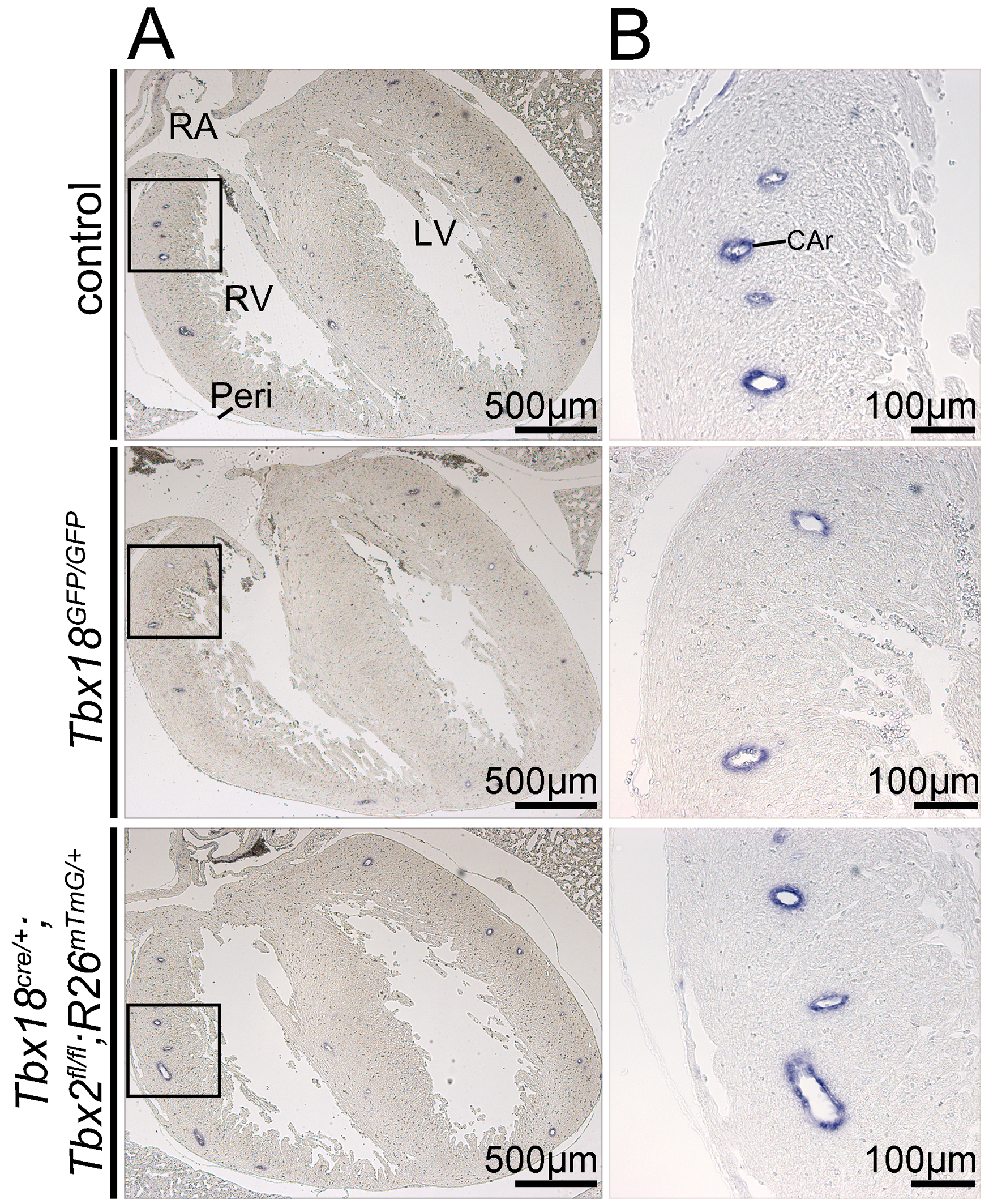

Supplement: S3 Fig — (A) In situ hybridization analysis of Myh11 expression on transverse sections of hearts of control, Tbx18GFP/GFP and Tbx18cre/+;Tbx2fl/fl;R26mTmG/+ mice at E18.5. As in control hearts, SMCs of the coronary arteries of both mutant hearts show Myh11 expression. (B) Shown are higher magnifications of the boxed areas in the right ventricle. Scale bars are as shown. CAr, coronary artery; LV, left ventricle; Peri, pericardium; RA, right atrium; RV, right ventricle. (TIF) [file pone.0156787.s003.tif]

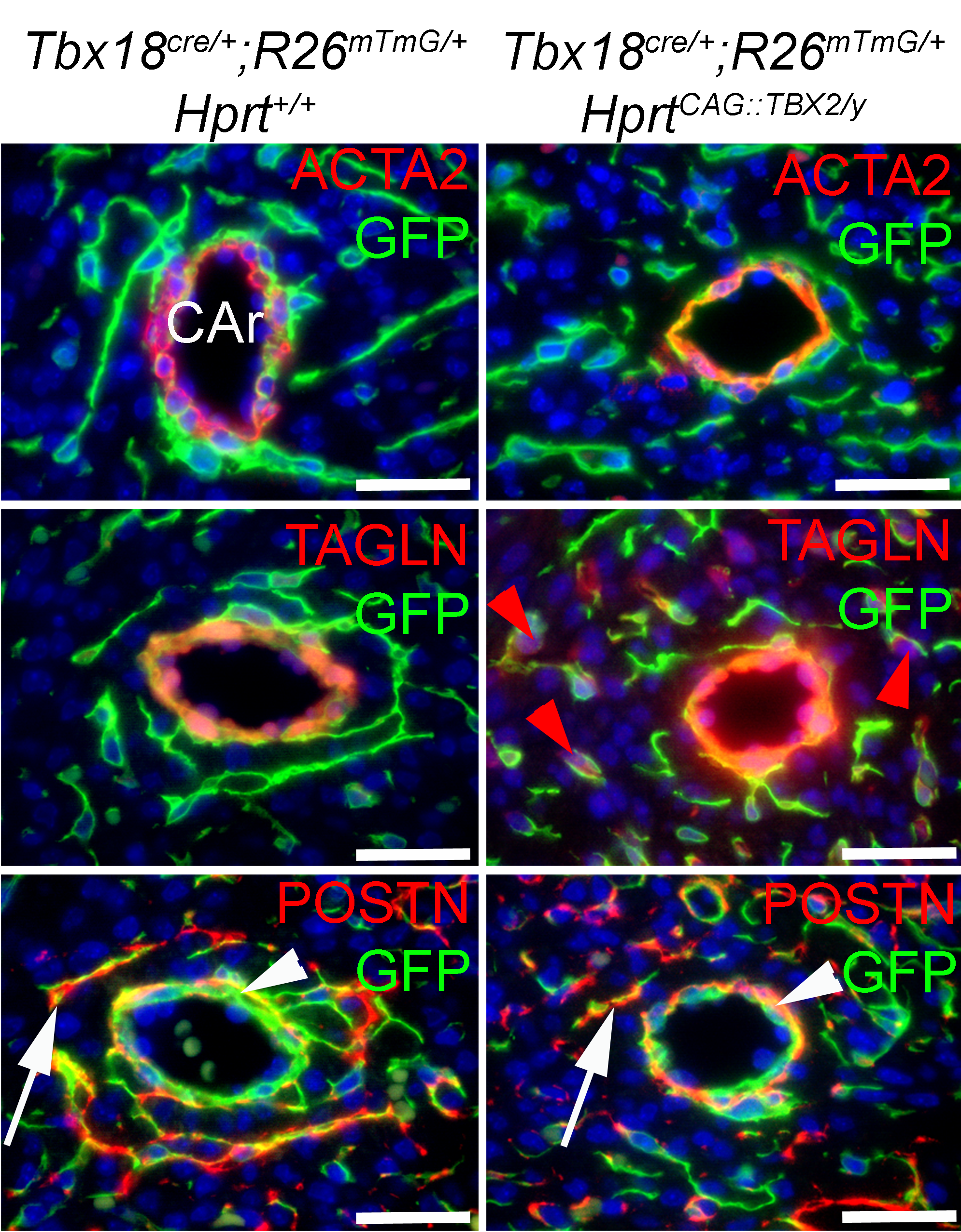

Supplement: S4 Fig — Epicardial cells stained for the lineage label GFP enter the myocardium of Tbx18cre/+;R26mTmG/+;HprtCAG::TBX2/y hearts as in Tbx18cre/+;R26mTmG/+;Hprt+/+ controls and contribute to coronary SMCs as indicated by double-immunofluorescence staining against the SMC marker proteins ACTA2 or TAGLN and GFP. Besides, intermyocardial epicardium-derived cells express TAGLN but not ACTA2 in Tbx18cre/+;R26mTmG/+;HprtCAG::TBX2/y hearts which was not observed in control hearts at E18.5 (red arrowheads). As in control hearts, epicardium-derived cells of mutant hearts contribute partially to cardiac fibroblasts as indicated by double staining for the epicardial lineage marker GFP and the fibroblast marker protein POSTN. The arrows point towards interstitial epicardium-derived fibroblasts whereas the arrowheads mark coronary fibroblasts derived from the epicardium. The number of analyzed specimen is two and the error bar represents 40 μm. CAr, coronary artery. (TIF) [file pone.0156787.s004.tif]
